# In Vivo Targeted MR Imaging of Endogenous Neural Stem Cells in Ischemic Stroke

**DOI:** 10.3390/molecules21091143

**Published:** 2016-08-29

**Authors:** Fang Zhang, Xiaohui Duan, Liejing Lu, Xiang Zhang, Xiaomei Zhong, Jiaji Mao, Meiwei Chen, Jun Shen

**Affiliations:** Department of Radiology, Sun Yat-Sen Memorial Hospital, Sun Yat-Sen University, Guangzhou 510120, Guangdong, China; xinxin110007@126.com (F.Z.); duanxiaohui-128@163.com (X.D.); luliejing@foxmail.com (L.L.); xiangz2007@163.com (X.Z.); zhxm07@126.com (X.Z.); canterburybells@126.com (J.M.); anyway90@163.com (M.C.)

**Keywords:** neural stem cells, adult, ischemic stroke, magnetic resonance imaging, monoclonal anit-CD15 antibody, iron oxide nanoparticles

## Abstract

Acute ischemic stroke remains a leading cause of death and disability. Endogenous neurogenesis enhanced via activation of neural stem cells (NSCs) could be a promising method for stroke treatment. In vivo targeted tracking is highly desirable for monitoring the dynamics of endogenous NSCs in stroke. Previously, we have successfully realized in vivo targeted MR imaging of endogenous NSCs in normal adult mice brains by using anti-CD15 antibody-conjugated superparamagnetic iron oxide nanoparticles (anti-CD15-SPIONs) as the molecular probe. Herein, we explore the performance of this molecular probe in targeted in vivo tracking of activated endogenous NSCs in ischemic stroke. Our study showed that intraventricular injection of anti-CD15-SPIONs could label activated endogenous NSCs in situ seven days after ischemic stroke, which were detected as enlarged areas of hypo-intense signals on MR imaging at 7.0 T. The treatment of cytosine arabinosine could inhibit the activation of endogenous NSCs, which was featured by the disappearance of areas of hypo-intense signals on MR imaging. Using anti-CD15-SPIONs as imaging probes, the dynamic process of activation of endogenous NSCs could be readily monitored by in vivo MR imaging. This targeted imaging strategy would be of great benefit to develop a new therapeutic strategy utilizing endogenous NSCs for ischemic stroke.

## 1. Introduction

Ischemic stroke is a major cause of death and the most common cause of adult-acquired disability [1,2]. Stem cell-based therapy using neural stem cells (NSCs), embryonic stem cells, mesenchymal stem cells, or induced pluripotent stem cells, has emerged as a new therapeutic strategy for ischemic stroke. Among them, transplantation of NSCs is a favorable choice because of their ability to self-renew, proliferate, and generate multiple cellular lineages [3,4,5]. However, using exogenous NSCs has several intrinsic technical and ethical issues. For example, a surgical procedure might be needed to inject cells accurately into host brains. The potential toxicity (e.g., carcinogenesis) may increase the complexity of cell therapies. In addition, cell survival and migration rely heavily on the timing and approach of stem cells delivery. Moreover, ethical issues may be raised from the use of fetal/embryonic cells [2]. Alternatively, endogenous neurogenesis was shown to be enhanced via endogenous NSCs proliferation, survival, and differentiation, which would provide another promising method for the treatment of stroke [1,4].

Endogenous NSCs mainly locate in the subventricular zone (SVZ) of the lateral ventricle and the subgranular zone (SGZ) in the dentate gyrus of the hippocampus and persist in the adult rodent brain [6,7]. The SVZ is the largest source of endogenous NSCs in the brain. NSCs formed in the SVZ commonly migrate along the rostral migratory stream (RMS) to the olfactory bulb (OB) where they differentiate into neurons [7]. It has been reported that endogenous NSCs are triggered to proliferate and migrate towards injured brain after ischemic stroke and, subsequently, differentiate into the phenotype of the destroyed cells [8]. To increase our understanding of the dynamic process of proliferation and migration of endogenous NSCs in the ischemic brain, development of methods with a capacity of in vivo tracking endogenous NSCs are highly desirable. In vivo visualization of the activation of endogenous NSCs would be of great benefit to the understanding of the plasticity of the SVZ under ischemic conditions and would be essential to utilize the therapeutic potential of endogenous NSCs in brain injury treatment.

Previously, in situ labeling by the direct injection of micron-sized particles of iron oxide (MPIO) or superparamagnetical iron oxide nanoparticles (SPIONs) into the lateral ventricle [9,10,11,12,13] or in the subventricular regions [10,11] have been found to be useful for in vivo tracking the migration of endogenous NSCs. Despite the unique advantage of this strategy, such in situ labeling strategy has several intrinsic limitations, e.g., significant image distortion of MPIO, non-targeting nature, and low labeling efficiency [14,15]. To overcome these shortcomings, a ferritin-based MR imaging reporter gene was used to non-invasively visualize the endogenous NSCs migration [15,16]. However, the low imaging sensitivity, non-targeting, and potential toxicity of the reporter gene hamper in vivo endogenous NSCs tracking applications [14]. Additionally, most of these studies investigated the in vivo tracking of migration of endogenous NSCs in normal rodent brains. Only a few studies have used this strategy to investigate endogenous NSCs in pathological conditions [13].

Previously, we have successfully realized in vivo targeted MR imaging tracking of endogenous NSCs in normal adult mice brains by using the molecular probe of anti-CD15 monoclonal antibody conjugated SPIONs (anti-CD15-SPIONs) [17]. In this study, we further explore the performance of this molecular probe in targeted tracking of activated endogenous NSCs after cerebral ischemia. To validate the targeted imaging capacity of this MR probe, cytosine arabinosine (Ara-C), a DNA synthesis inhibitor to prevent cell proliferation [18,19], was used to inhibit endogenous NSCs proliferation induced by ischemic stroke. The aim of this study is to investigate the feasibility of in vivo targeted MR imaging of activated endogenous NSCs in ischemic stroke brains by using anti-CD15-SPIONs as the imaging probe.

## 2. Results

### 2.1. In Vivo MR Imaging of Activated NSCs in Ischemic Stroke

**Figure 1:**
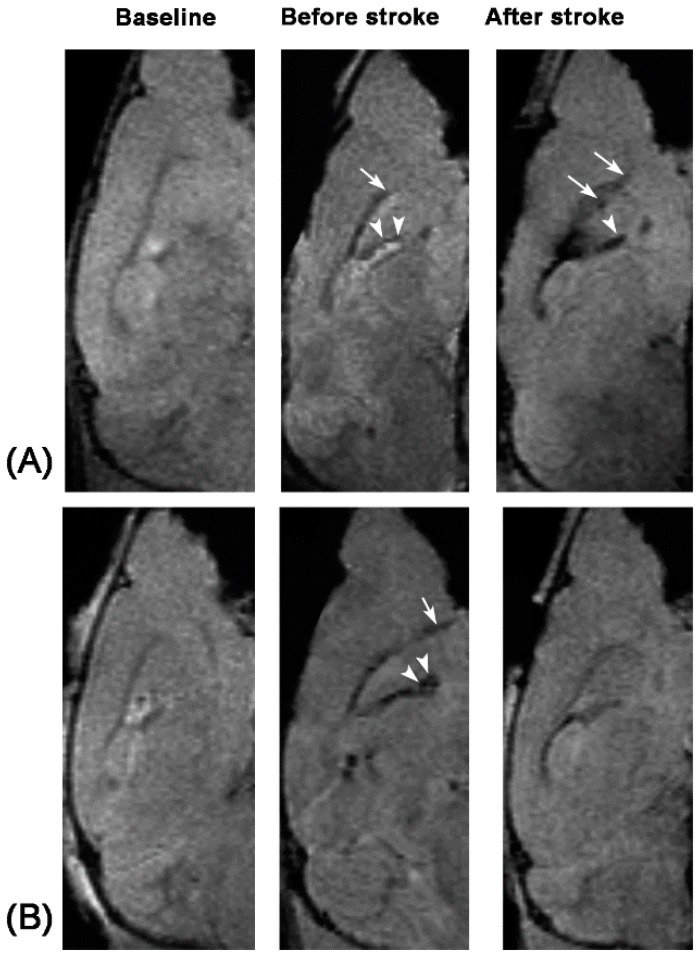
Serial in vivo MR images of endogenous NSCs from a pure stroke mouse and a stroke mouse treated with Ara-C. Compared with baseline sagittal T2*-weighted images, after intraventricular injection of anti-CD15-SPIONs, linear hypointense signal (arrows), and spotty hypointense signals (arrowheads) appear in the beginning of RMS and SVZ both in the mouse with pure stroke group (**A**) and the mouse with Ara-C treatment (**B**) before induction of stroke. Eight days after stroke, the area of hypointense signal increases in the pure stroke mouse (**A**), while it diminishes in the stroke mouse treated with Ara-C (**B**).

**Figure 2:**
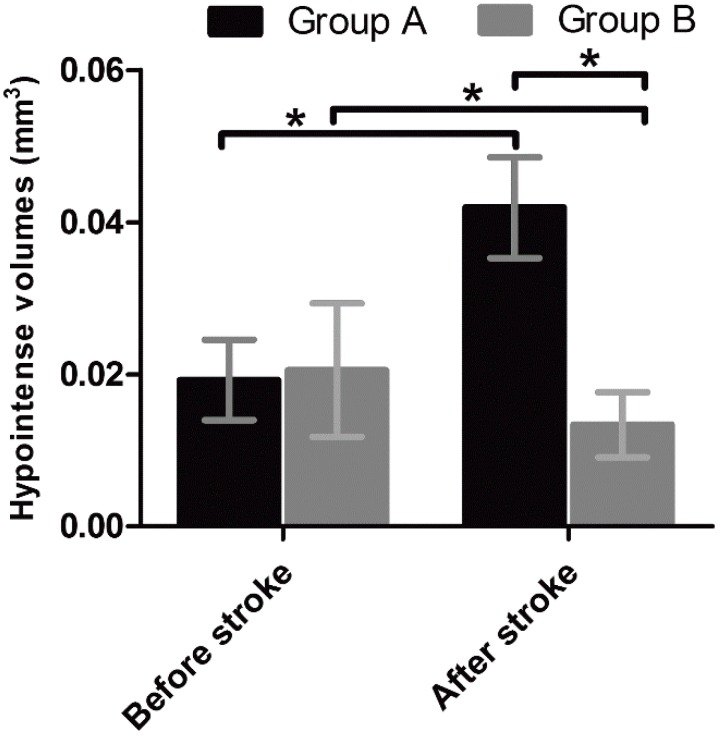
Volumes of hypointense signals in the SVZ and RMS in pure stroke mice and stroke mice treated with Ara-C. Graphs show the volumes of hypointense signal measured in the SVZ and RMS on T2*-weighted MR images in pure stroke mice (group A) and stroke mice treated with Ara-C (group B) before induction of stroke and eight days after the stroke. * *p* < 0.05.

To determine the capacity of in vivo targeted MR imaging of activated NSCs, 12 mice received intraventricular injection of anti-CD15-SPIONs before and seven days after stroke. One day after injection of the imaging probe, the SVZ and the beginning of the RMS were detected as spotty and linear hypointense signals on T2- and T2*-weighted imaging before stroke. Eight days after stroke, these hypointense areas were obviously enlarged (Figure 1A). The volume of hypointense areas was measured before and eight days after stroke. The hypointense volume in SVZ and the beginning of the RMS after eight days of stroke was significantly higher than that before stroke (*p* < 0.01) (Figure 2).

### 2.2. In Vivo MR Imaging of Inhibited NSCs by Ara-C

To further verify the presumption of targeted imaging capacity of anti-CD15-SPIONs, eight stroke mice received intraventricular injection of Ara-C immediately after the establishment of stroke for seven days. Before stroke, the SVZ and the beginning of the RMS in these animals were also detected as spotty and linear hypointense signals on T2- and T2*-weighted imaging. After seven days of Ara-C infusion, hypointense signals in the SVZ and RMS in stroke mice were almost no longer discernable (Figure 1B). The volume of hypoinense signals in the SVZ and the beginning of the RMS on T2- and T2*-weighted imaging were measured before stroke and at eight days after stroke in animals treated with Ara-C. The hypointense volume was significantly lower than that before stroke (*p* < 0.05) (Figure 2). In addition, the volume of hypointense signal measured before stroke was similar between pure stroke animals and animals treated with Ara-C, while it was significantly lower in stroke animals treated with Ara-C than that in pure stroke animals after eight days following ischemic stroke (*p* < 0.05) (Figure 2). These findings confirmed the specifically-targeted imaging of NSCs by anti-CD15-SPIONs.

### 2.3. Infarct Volume

**Figure 3:**
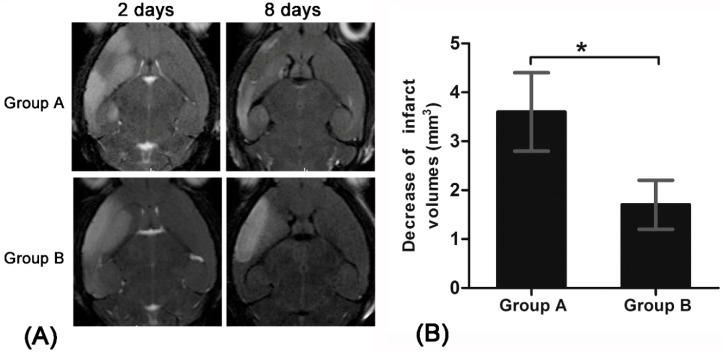
Infarct volumes in pure stroke mice and stroke mice treated with Ara-C. T2-weighted MR images show hyperintense signal of the cerebral infarction on T2-weighted MR images (**A**); Graphs show infarct volume decrease measured in pure stroke mice (group A) and stroke mice treated with Ara-C (group B) between 2 days and 8 days after the stroke (**B**). * *p* < 0.05.

To observe the effect of endogenous NSCs on cerebral infarction, the infarct volume was measured in stroke animals treated with Ara-C and pure stroke animals at two and eight days after stroke. In pure stroke animals, the infarct volume was declined during the period of one to eight days after stroke (*p* < 0.05). No such significant change was found in stroke animals treated with Ara-C (*p* > 0.05). A much greater decrease of infarct size was found in pure stroke animals than in Ara-C injection animals (*p* < 0.05) (Figure 3).

### 2.4. Histology

**Figure 4:**
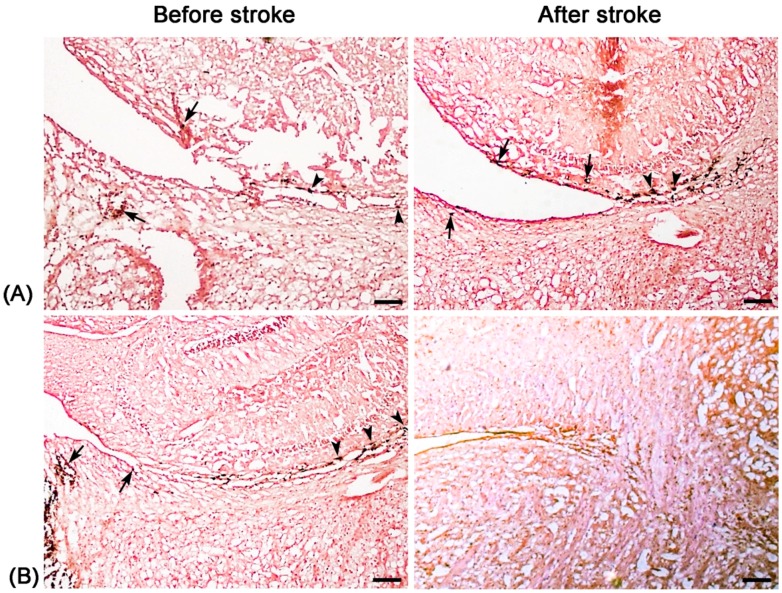
Histologic assessment of the distribution of molecular probes. Representative micrographs show that after intraventricular injection of anti-CD15-SPIONs, DAB-enhanced Prussian blue staining reveals that positively stained anti-CD15-SPIONs (dark brown) are localized around cells and extracellular matrix within SVZ (arrows) and RMS (arrowheads) before stroke in the pure stroke mouse (**A**) and the stroke mice with Ara-C treatment (**B**); Eight days after stroke, the number of positive particles in the pure stroke mouse increases obviously (**A**) but remarkable decrease is found in the stroke mouse after administration of Ara-C (**B**). Bars = 50 μm.

**Figure 5:**
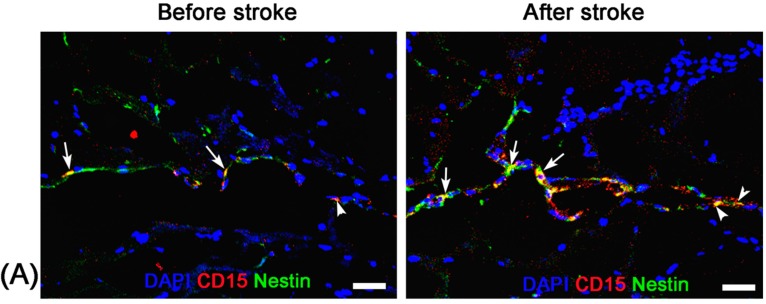

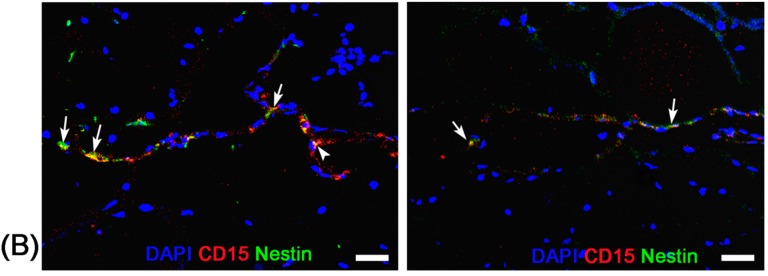
Immunofluorescence studies of the activation of endogenous NSCs. Representative anti-CD15 (red) and anti-Nestin (green) immunofluorescence staining micrographs show the presence of CD15^+^Nestin^−^ and CD15^+^Nestin^+^ cells in the SVZ (arrows) and beginning of RMS (arrowheads) before the establishment of stroke both in pure stroke mouse (**A**) and Ara-C-treated mouse (**B**). There are abundant CD15^+^Nestin^−^ and CD15^+^Nestin^+^ cells in the SVZ and RMS in pure stroke animals eight days after stroke. Almost no CD15^+^Nestin^−^ or CD15^+^Nestin^+^ cells are present in stroke animals after administration of Ara-C for seven days. Bars = 25 μm.

DAB-enhanced Prussian blue staining shown that more positively stained particles within the SVZ and RMS were found in pure stroke animals at 8 days after ischemic stroke compared with that before stroke. Only a few positive particles within the SVZ and RMS were found in stroke animals with administration of Ara-C inhibition for seven days (Figure 4). Immunofluorescence staining revealed that a small amount of CD15^+^Nestin^−^ and CD15^+^Nestin^+^ cells were located in the SVZ and the beginning of RMS before the establishment of stroke both in pure stroke and Ara-C-treated animals. Eight days after stroke, abundant CD15^+^Nestin^−^, and in particular CD15^+^Nestin^+^ cells were found in the SVZ and RMS in pure stroke animals, but those CD15^+^Nestin^−^ or CD15^+^Nestin^+^ cells had almost disappeared in stroke animals treated with Ara-C (Figure 5). The location and distribution of the positively-stained SPIONs, CD15^+^Nestin^−^ or CD15^+^Nestin^+^ cells were spatially correlated with the hypointense signals detected by MR imaging.

## 3. Discussion

Our study showed that anti-CD15-SPIONs could specifically accumulate around a subpopulation of endogenous NSCs with a CD15 phenotype in the SVZ and RMS after intraventricular injection in ischemic stroke. Upon this targeted binding, the distribution of endogenous NSCs in native state could be detected by MR imaging. Ischemic injury could activate endogenous NSCs to proliferate in the SVZ and migrate along the RMS [2,13]. Using anti-CD15-SPIONs as imaging probe, such dynamic process could be readily monitored by in vivo MR imaging. The treatment of Ara-C could block activation of endogenous NSCs in ischemic stroke. This inhibition could be tracked by MR imaging.

The generation of new neurons in the adult brain is spatially restricted to the SVZ surrounding the lateral ventricles and the SGZ of the dentate gyrus [6,7]. SVZ is the largest source of endogenous NSCs that can proliferate, migrate, and differentiate into neurons, astrocytes, and oligodendrocytes by various normal and pathological stimuli [6,20]. Indeed, increased proliferation, migration, and differentiation of endogenous NSCs in the SVZ has been observed in animal stroke models and even in stroke patients [3,4,21]. The activation of endogenous NSCs might contribute to the spontaneous recovery that occurs even without any treatment after brain stroke [2]. Mechanisms for the beneficial effect of endogenous NSCs in stroke involve producing neurotrophic factors, regulating the inflammatory environment, producing proangiogenic complexes and secreting factors promoting synaptic plasticity [2,20]. In our study, a significant decrease of infarct size over time were found in pure stroke animals but not in Ara-C treated animals. This finding may be explained by spontaneous recovery induced by the activation and proliferation of endogenous NSCs after stroke in our pure stroke animals [2], while the activation and proliferation of endogenous NSCs were inhibited and blocked in Ara-C treated animals [18,19]. In addition, proliferation and migration of endogenous NSCs in ischemic stroke were visualized by in vivo MR imaging with the use of a targeted imaging probe, which was similar with previous studies where nonspecific iron oxide nanoparticles were used [13,20]. 

MR imaging is a unique tool that has been widely used to non-invasively monitor the cell trafficking in vivo. At present, direct injection of iron oxide particles (from nanometer to micrometer size range) into the lateral ventricle [9,10,11,12,13] or in the subventricular regions [10,11] is the most common method for in situ labeling endogenous NSCs. After a portion of the injected iron oxide particles taken up by endogenous NSCs and carried away toward the OB, the dynamic signal intensity changes could be visualized by MR imaging, implying the success of this strategy for in situ cellular MR imaging. However, the labeling efficiency of this labeling protocol is relatively low, and only a small fraction of subpopulation cells will be labeled with the iron oxide particles. A previous study has showed that the total MPIO-labeled cells in the SVZ/RMS varied from 10% to 35%. Of these MPIO-labeled cells, only 28% were labeled on neurons and these labeled neurons decreased to 11% in the OB [10]. Most importantly, the cellular uptake of iron oxide particles in these approaches is rather non-specific and non-targeted. For example, MPIOs were not only inside the NSCs, but also in microglia, ependymal cells, and oligodendrocyte progenitor cells after direct injected into the lateral ventricle [9,10,22]. The detected signal intensity on MR images refers only to the iron oxide particles, but not to the certain cell type or viability of the labeled cells, resulting in the non-specific tracking on MR imaging. Moreover, direct injecting iron oxide particles in the lateral ventricles of the brain may induce considerable susceptibility effects and signal distortion on MR imaging around the region of injection, which may hamper the visualization of the RMS near the injection site and allow only for the detection of cells migrating away from the injection site. A recent approach using ferritin-based reporter gene was successfully used to monitor the migration of endogenous NSCs progeny to the OB at 30 weeks post injection [15,16]. However, this ferritin-based reporter gene system lacked reliable sensitivity to demonstrate the longitudinal migration of endogenous NSCs in vivo [16]. In addition, ferritin-like hypointense contrast on MR imaging may be elicited by viral vector systems themselves and may influence the iron homeostasis of host brain [15]. Thus, this labeling strategy must be carefully evaluated and these problems must be resolved before its clinical applications. 

Unlike nonspecific SPIONs/MPIO or ferritin-based reporter gene, in this study we used targeting SPIONs with anti-CD15 mAb as the ligand for in vivo MR imaging of endogenous NSCs on the basis of specific expression of CD15 antigen on the surface of distinct pools of NSCs in adult mice. After intraventricular delivery of these anti-CD15-SPIONs, endogenous NSCs in the SVZ and RMS were detected as hypointense signals on MR imaging before cerebral infarction and the volume of these hypointense signals obviously increased at eight days after stroke. Histology confirmed that these nanoparticles are targeted to cell surface of CD15-postive NSCs in SVZ and migrating to OB along the RMS after ischemic stroke. These results indicated that targeted MR imaging of activated endogenous NSCs in ischemic stroke was successfully achieved by using CD15-conjugated SPIONs as imaging probe. Compared to direct injection of MPIO or SPIONs complex, the use of CD15-targeted SPIONs has several advantages. First, due to anti-CD15 mAb specially recognizing the cell surface antigen CD15, the molecular probe could specially label CD15-positive NSCs and allow for specifically MR imaging monitoring of the migration of subpopulation of NSCs, reflecting the actual change in NSCs plasticity. Moreover, unlike internalization of larger MPIOs or SPIONs complex, cellular surface binding of these biodegradable nanoparticles would be more safety for in vivo cellular imaging as the intact biological behavior of labeled cells would be less affected. In addition, the image distortion and susceptibility artifact of SVZ in our study was negligible because of the small size and a small volume of the nanoparticles used, allowing clear visualization of entire SVZ and RMS even near the injection site.

To date, the majority of studies on MR imaging of endogenous NSCs were performed in naive and healthy animals. Only a few studies have used in situ labeling strategy to monitor the endogenous NSCs migration in abnormal conditions, such as ischemic stroke [13], multiple sclerosis [23], and tumor [24]. Previously, after intraventricular injection of MPIOs, endogenous NSCs in the SVZ in hypoxic-ischemic animal brain were found to mainly migrate towards the hypoxic-ischemic lesion sites and the migratory pathway of SVZ/RMS to OB could be observed for two weeks, as revealed by MR imaging [13]. Our results revealed the proliferation of endogenous NSCs but no migration towards the infarction lesion. This might be assumed to a short-term follow-up (eight days after ischemic stroke) or a relatively low imaging sensitivity of SPIONs than MPIOs. Another possible explanation is that only CD15-positive subpopulation of endogenous NSCs were labeled. The number of CD15-positive NSCs is far less than non-specifically labeled cells using MPIOs such as not only neural stem/progenitor cells but also newly-generated cells including neuroblasts, astrocytes, astrocytes-like progenitor cells and mature neurons.

To exclude false-positive hypointense signals induced by non-specific binding with NSCs from degraded anti-CD15-SPIONs, inflammation, or iron accumulation due to hemorrhage or degrading, we used Ara-C to inhibit the proliferation of endogenous NSCs as a negative control. Ara-C is an anti-mitotic agent that could inhibit the SVZ neurogenesis [19]. When Ara-C was infused into the cerebral lateral ventricle during the early phase of ischemic stroke, brain damage-induced NSCs proliferation and migration in the SVZ can be inhibited and blocked, resulting in increased neuron loss and worsened neurological deficits [18,19]. In our study, with use of Ara-C infusion, the hypointense signals in the SVZ and RMS were almost eliminated after injection of anti-CD15-SPIONs. Histology confirmed a remarkable decrease in the number of accumulated molecular probes and CD15-positive NSCs in these regions. These findings corroborated the specific labeling thereby targeted imaging of endogenous NSCs.

There are several limitations in our study. First, the potential host immunological reaction originated from the introduction of extrinsic antibody must be considered carefully before its clinical application. In our study, a homologous murine monoclonal antibody was conjugated to SPIONs with which the immunological reaction might be alleviated. With the progress of molecular biology, utilization of a specific single-chain Fv antibody would potentially overcome this disadvantage through decreased immunogenicity, and favorable pharmacokinetics profiles [25]. Second, potential toxicity associated with the accumulation of SPIONs in the cytoplasm should also be further carefully evaluated. Third, only immunohistological staining for CD15 was performed in histology, several other neural cell phenotypes, including ependymal cells, neuroblasts, oligodendrocytes, and microglia, as well as the quantitative analysis of labeled cells were not attempted in this study. However, a previous study had characterized a CD15-positive cell population in the SVZ and indicated little overlap with other phenotypes [26]. Lastly, only CD15 was used to targeting ligand to label the endogenous NSCs. Since not all adult endogenous NSCs exclusively express CD15, and CD15 is also expressed by a small amount of subpopulations of astrocytes, tanycytes, and neurons in the adult mouse brain [26,27]. A CD15-conjungated probe cannot specifically label all of the adult endogenous NSCs but just a CD15-positive subpopulation. However, previous study showed that CD15 antigen is a cellular surface ligand that was abundantly expressed in adult endogenous NSCs in the SVZ regions, indicating that the use of CD15 as a targeting ligand is suitable for targeted in vivo imaging most of adult mouse NSCs [26]. Combining CD15 ligand with other additional specific markers would improve the capability of targeted imaging via a nanoprobe, which needs further investigation.

## 4. Materials and Methods

### 4.1. Animals

All experimental procedures were in accordance with the guidelines for the care and use of laboratory animals and the ethical review process of our institution and were approved by the Institutional Animal Care and Use Committee of Sun Yat-Sen University. A total of 45 adult 8–10 week-old C57BL/6J mice (weighing 20–25 g) were obtained from the Laboratory Animal Center of Sun Yat-Sen University. All animals were bred in a standard animal facility with 12 h on/off light conditions and allowed standard food and water ad libitum. The mortality associated with the stroke induction and/or injection procedures was 13.3% (*n* = 6). Nineteen mice with failed ischemic stroke mode (*n* = 11) and unsatisfied stereotactical injection (*n* = 8) were excluded. The remaining 20 mice were included in this study. During surgical and injection procedures, a heating pad and an overhead lamp was used to maintain the temperature at approximately 37 °C. After surgery, the surgical wound was checked for infection every day, and gentamycin was administered when there was evidence of wound infection. Glucose water were administered if there was evidence of decreased appetite.

### 4.2. Imaging Probes

SPIONs conjugated with a monoclonal rat anti-mouse CD15 IgM antibody (anti-CD15-SPIONs; Miltenyi Biotech, Bergisch Gladbach, Germany) are commercially available and were used as the molecular imaging probe. These iron oxide particles are composed of a biodegradable, non-toxic dextran-based ferromagnetic matrix, and their nominal overall mean diameter was approximately 50 nm with a diameter of 30 nm for the magnetic core. There were typically 10–200 antibody molecules per particle. Their magnetic properties and capacity of specific binding with NSCs have been described in our previous study [17].

### 4.3. Experimental Stroke and Design

Before being subjected to ischemic stroke, all animals underwent baseline MR imaging before and after intraventricular injection of anti-CD15-SPIONs. As our previous study showed that the subpopulation of NSCs in the anterior SVZ and the beginning of RMS could be in situ labeled by anti-CD15-SPIONs and be visualized at one day after the injection [17], MR imaging after intraventricular injection of anti-CD15-SPIONs was performed at one day after the injection to observe normal SVZ and RMS. After baseline MR imaging, acute cerebral ischemic stroke was established by direct intraluminal suture occlusion of the right middle cerebral artery, as described previously [28]. In brief, after anesthesia with peritoneal injection of 1% pentobarbital sodium (6 mL/kg), a nylon poly-l-lysinecoated 4-cm suture with a blunt head (model 2636; Sunbio Biotech, Beijing, China) was inserted 1.2 cm into the internal carotid artery via the proximal external carotid artery. After 20 min occlusion, blood reperfusion was allowed by withdrawal of the suture. Two days after middle cerebral artery occlusion, animals underwent MR imaging again to confirm the establishment of cerebral infarction. Twenty stroke mice were randomly divided into two groups: pure stroke group (*n* = 12) and Ara-C group (*n* = 8). Since our previous study revealed that the in situ labeling of endogenous NSCs persisted at most seven days after the injection [17], animals in the pure stroke group received the second injection of anti-CD15-SPIONs at seven days after stroke to exclude the interference of potentially retained anti-CD15-SPIONs initially injected. In Ara-C group, immediately after stroke, animals received slow infusion of 90 μL 2% Ara-C into the contralateral cerebral lateral ventricle to inhibit endogenous NSCs proliferation and migration induced by brain ischemia, as previously described [10]. The infusion of Ara-C was performed at pumping rate of 0.5 μL/h for seven days by using ALZET^®^ micro-osmotic pump system (Model 1007D, DURECT Corporation, Cupertino, CA, USA). After that, animals received the second episode of injection of imaging probe. One day later, brain MR imaging was performed to detect the activation and inhibition of SVZ or RMS after stroke.

### 4.4. Surgical Injection

Imaging probe, anti-CD15-SPIONs were stereotactically injected into the left cerebral lateral ventricle at a dosage of 7 μL per animal. Anti-CD15-SPIONs were injected into the anterior portion of the lateral ventricles on right side with a stereotaxic coordinate: 0.95 mm lateral to bregma, 0.02 mm rostral to bregma and 2.6 mm deep from the pial surface by the same operator (F.Z., with five years of experience with microsurgical procedures). The anti-CD15-SPIONs was slowly injected with a constant rate of 0.5 μL/min using a 28 gauge needle (NanoFil; World Precision Instruments, Sarasota, FL, USA) mounted on a microinjector. After injection, the needle was left in the place for 5 min and then slowly withdrawn. 

### 4.5. In Vivo MR Imaging

Brain MR imaging was acquired on a 7.0 T micro-MR scanner (PharmaScan, Bruker, Ettlingen, Germany) with a 23-mm mouse brain coil. After anesthetized by isoflurane (1%–1.5% at 0.8–1.0 L/min air flow via a nose cone) with respiratory monitoring, mice were positioned in a plastic holder with a stereotaxic head-frame. The pulse sequences included axial, coronal and sagittal two-dimensional T2*-weighted fast low angle shot gradient echo sequence (TR/TE = 400/3.5; flip angle= 30°; matrix = 256 × 256; field of view = 30 mm; number of signals acquired = 8; section thickness/gap = 0.5 mm/0) and two-dimensional T2-weighted turbo rapid acquisition with relaxation enhancement sequence (TR/TE = 6000/60; matrix = 256 × 256; field of view = 30 mm; number of signals acquired = 6; section thickness/gap = 0.5 mm/0). To observe the dynamic change of NSCs and cerebral infraction, the volume of hypointense signals in SVZ-RMS was measured on T2*-weighted imaging and the infarct volume of brain was measured on T2-weighted imaging using same technique of region of interest with ImageJ software (National Institutes of Health, Bethesda, MD, USA), as previous described [28].

### 4.6. Histology

Before stroke and eight days after stroke, two mice from each group were randomly chosen at each time point and sacrificed by anaesthetic overdose immediately after the completion of MR imaging. After transcardial perfusion with saline and further 4% paraformaldehyde, animal brains were removed, post-fixed in 10% paraformaldehyde overnight and then cryoprotected in 30% sucrose solution. Contiguous 5-μm thickness sagittal sections were cut and processed for diaminobenzide (DAB)-enhanced Prussian blue staining to determine the distribution of nanoparticles. Moreover, immunofluorescence staining for CD15 and Nestin was performed to observe the activation of NSCs and verify the presence of anti-CD15 SPIONs around NSCs. 

For DAB-enhanced Prussian blue staining, sections were incubated with Prussian blue solution (15% hydrochloride and 10% potassium ferrocyanide (II) trihydrate) for 30 min, and reacted with unactivated and activated (containing 0.03% hydrogen peroxide) 0.014% diaminobenzide for 15 min, and then counterstained with nuclear fast red. For immunofluorescence staining, adjacent sections were incubated with the primary antibodies against CD15 (1:200; BD Pharmingen, San Jose, CA, USA) and Nestin (1:200; Chemicon, Temecula, CA, USA) overnight at 4 °C. The secondary antibody FITC-conjugated anti-mouse IgM (Chemicon) and Cy3-conjugated anti-mouse IgG (Chemicon) was applied for 30 min at room temperature. DAPI (1:1000; Sigma-Aldrich, St. Louis, MO, USA) was applied to label the nuclei. Fluorescence images were observed on a fluorescence microscope (TE2000-U; Nikon Company, Tokyo, Japan).

### 4.7. Statistical Analysis

Data were expressed as mean ± SD, unless indicated otherwise. The hypointense volume measured before stroke and 8 days after stroke and infarct volume measured 2 days and 8 days after stroke were analyzed by using a pairwise *t* test. The comparisons of hypointense volume and infarct volume between two groups at each time point by using two independent samples *t* test. Statistical analysis was performed with SPSS 18.0 software for windows (version 18.0; SPSS Inc., Chicago, IL, USA). *p* < 0.05 was considered to indicate a statistically significant difference.

## 5. Conclusions

Our study demonstrated that ischemic stroke could activate the proliferation and migration of endogenous NSCs. The expansion and migration of endogenous NSCs in ischemic stroke could be successfully and readily monitored by in vivo targeted MR imaging after intraventricular delivery of anti-CD15 mAb-conjugated SPIONs. This non-invasive imaging strategy would facilitate the study of neurogenesis mechanisms involving endogenous NSCs and would be of great benefit to develop new therapeutic strategies utilizing the full potential of endogenous NSCs for ischemic stroke or other central nerve system injuries.

## Abbreviations

The following abbreviations are used in this manuscript:

NSC	neural stem cell
SVZ	subventricular zone
SGZ	subgranular zone
RMS	rostral migratory stream
OB	olfactory bulb
MPIO	micron-sized particles of iron oxide
SPION	superparamagnetical iron oxide nanoparticle
Ara-C	Cytosine Arabinosine
